# Religious and secular moral orientations as potential moderators of the association between mental wellbeing and future anxiety

**DOI:** 10.3389/fpsyg.2026.1952527

**Published:** 2026-10-22

**Authors:** Abdurrahman Akbolat

**Affiliations:** Department of Philosophy and Religious Studies, Faculty of Theology, İzmir Kâtip Çelebi University, İzmir, Türkiye

**Keywords:** future anxiety, mental wellbeing, moderation analysis, religious moral orientation, secular moral orientation, university students

## Abstract

**Background:**

Mental wellbeing is inversely associated with future anxiety, but it remains uncertain whether moral reference systems condition the strength of this association. This study examined religious moral orientation (RMO) and secular moral orientation (SMO) as potential moderators.

**Methods:**

A convenience sample of 400 university students in Türkiye completed self-report measures of mental wellbeing, future anxiety, RMO, and SMO. Analyses involving future anxiety used 391 complete cases. RMO and SMO were scored as distinct subscales and were not summed. Separate moderated regressions constituted the primary analyses; bootstrap, HC3-robust, joint-model, and covariate-adjusted estimates were examined as sensitivity analyses.

**Results:**

Higher mental wellbeing was associated with lower future anxiety. The mental wellbeing × RMO interaction was negative under normal-theory estimation (*p* = 0.003), but the percentile-bootstrap 95% confidence interval included zero and the separate-model HC3 result was just above the conventional threshold (*p* = 0.0503). The mental wellbeing × SMO interaction was not significant in the primary model (*p* = 0.078). Neither the unstandardized (*p* = 0.877) nor fully standardized (*p* = 0.553) joint-model contrast indicated that the interaction coefficients differed.

**Conclusion:**

The inverse association between mental wellbeing and future anxiety may vary with moral orientation, but evidence for RMO as a moderator was estimation-sensitive and the data did not establish distinct moderating effects for RMO and SMO. The findings are preliminary because the design was cross-sectional, the sample came from one university, and the interaction effects were small.

## 1 Introduction

Future anxiety can be described as an emotional state characterized by fear, worry, and uncertainty regarding potentially negative developments in one's long-term future (Zaleski, 1996). Amid interconnected global and local crises, uncertainty has increasingly become a defining feature of contemporary life, intensifying concerns about the future (United Nations Development Programme, 2022). Persistent future anxiety may interfere with individuals' sense of personal agency and their capacity to pursue future-directed goals (Bandura, 2001). Research has consistently associated heightened future anxiety with poorer psychological adjustment, including elevated levels of depression, stress, and psychological distress, particularly under conditions of prolonged uncertainty (Öztekin et al., 2025). This pattern highlights the importance of identifying psychological resources that may buffer the adverse effects of future anxiety and help explain why some individuals adapt more successfully than others under conditions of pervasive uncertainty (Zimmermann et al., 2020).

Mental wellbeing is a broad indicator of positive mental functioning that includes both hedonic experiences and eudaimonic functioning (Tennant et al., 2007; van Agteren et al., 2021). The present study operationalizes this construct with the Warwick–Edinburgh Mental WellBeing Scale (WEMWBS), which combines positively worded indicators of feeling and functioning in a single total score. Ryff's psychological wellbeing framework is relevant background for understanding eudaimonic functioning (Ryff, 1989, 2014; Ryff and Keyes, 1995), but the WEMWBS does not assess Ryff's six dimensions separately. Accordingly, the measured construct is termed mental wellbeing throughout this article, and no dimension-specific claims are made.

Although higher mental wellbeing is generally associated with more adaptive functioning under stress and uncertainty, the strength of its association with future anxiety may vary across individuals. Moral orientation is one possible source of such variation because moral and existential reference systems can shape how people interpret uncertainty and regulate behavior (Haidt, 2001; Graham et al., 2013; Yapici, 2007). However, the literature does not support a simple assumption that religious worldviews are inherently healthier than non-religious ones. A curvilinear pattern has been reported in which confidently religious and confidently non-religious people show more favorable wellbeing than those with less certain positions (Galen and Kloet, 2011). Research focused specifically on non-religious populations likewise emphasizes secular meaning systems, identity clarity, social context, and community rather than non-belief itself as determinants of adjustment (Galen, 2018; Speed and Hwang, 2019; Uzarevic and Coleman, 2021). Among Polish non-believers, (Łowicki and Barker 2025) found comparable overall wellbeing among Catholics and atheists, lower scores among religiously unaffiliated participants, and positive associations of self-belief clarity, adaptive emotion regulation, and social support with wellbeing or meaning. Taken together, this evidence suggests that coherence and stability of a worldview may matter more than whether its content is religious or secular.

One way of conceptualizing these relatively stable individual differences is through moral orientation. Following (Koçak 2025), moral orientation refers to individuals' tendency to rely predominantly on either religious or secular reference systems when making moral judgments and regulating their behavior. Religious moral orientation (hereafter RMO) reflects a tendency to evaluate moral issues primarily through religious beliefs and transcendent sources, whereas secular moral orientation (hereafter SMO) reflects greater reliance on reason, human experience, and ethical principles that are not necessarily grounded in religious authority (Audi, 2000; Koçak, 2025). These orientations do not refer to moral behavior itself; rather, they describe the normative reference systems individuals draw upon when interpreting morally relevant situations and deciding how to act. Importantly, endorsing a secular moral orientation should not be interpreted as indicating the absence of religiosity. Individuals may rely on both religious and secular moral reference systems, although the relative prominence of each orientation may vary across persons and situations (Koçak, 2025). From this perspective, moral orientation is better understood not as a direct indicator of religious commitment but as a psychological tendency to rely more heavily on particular normative reference systems. This distinction may help explain why individuals with similar levels of mental wellbeing do not necessarily respond to future uncertainty in the same way.

Although empirical research directly examining moral orientation remains limited, conceptually related constructs provide indirect evidence for its potential relevance to psychological adaptation. Moral identity, which refers to the extent to which moral values are central to individuals' self-concept, has consistently been linked to higher levels of mental wellbeing and adaptive functioning. A recent meta-analysis of 27 studies found that stronger moral identity was associated with greater emotional wellbeing, including higher levels of happiness, life satisfaction, self-esteem, and meaning in life (Goering et al., 2024). In addition, moral identity has been shown to contribute indirectly to subjective wellbeing through stronger and more coherent identity commitments (Cui et al., 2021). Longitudinal evidence further indicates that moral identity uniquely predicts mental wellbeing across adolescence and early adulthood, even after accounting for other positive developmental characteristics (Lawford et al., 2023). Although moral identity and moral orientation are conceptually distinct constructs, these findings suggest that relatively stable moral reference systems may play an important role in psychological adaptation under conditions of uncertainty.

Recent research has increasingly shifted from documenting the detrimental consequences of future anxiety toward identifying the psychological resources and contextual factors that support adaptive functioning under conditions of uncertainty. Resilience, subjective wellbeing, sense of coherence, identity-related processes, and psychological flexibility have all been associated with lower levels of future anxiety and better psychological adjustment (Lorini et al., 2023; Dalmiş et al., 2025; Ingrassia et al., 2025; Sun, 2026). Evidence from the psychology of religion similarly suggests that religiosity may buffer the adverse psychological consequences of uncertainty (Türk et al., 2025). Research in moral psychology has also linked moral identity and moral foundations to various indicators of mental wellbeing (Goering et al., 2024; Miwa, 2025). Together, this body of research suggests that psychological adaptation under conditions of uncertainty is shaped by multiple, interconnected psychological processes. However, studies on mental wellbeing, moral orientation, and future anxiety have largely developed independently, leaving unanswered whether the moral reference systems individuals rely upon influence the inverse association between mental wellbeing and future anxiety.

The present study therefore tests whether moral orientation conditions the inverse association between mental wellbeing and future anxiety. RMO was expected to be associated with a steeper inverse relation because religious frameworks may provide stable meaning, order, and interpretive guidance under uncertainty. The direction and mechanism of an SMO interaction are less clearly established: secular reference systems may also provide coherence and meaning, but their relevance may depend more strongly on identity clarity and social context. The SMO analysis was therefore designated exploratory. The following hypotheses were examined:

H1. Mental wellbeing will be negatively associated with future anxiety.
H2. RMO will moderate the association between mental wellbeing and future anxiety, such that the inverse association will be stronger at higher RMO.
H3 (exploratory). SMO will moderate the association between mental wellbeing and future anxiety.

## 2 Materials and methods

### 2.1 Participants

Participants were 400 university students (138 men, 34.5%; 262 women, 65.5%) enrolled at Izmir Kâtip Çelebi University and recruited using convenience sampling. Participants ranged in age from 17 to 55 years (*M* = 23.22, SD = 4.90). Faculty representation was highly unbalanced: Theology (Divinity), *n* = 223; Social and Human Sciences, *n* = 100; Medicine, *n* = 1; Health Sciences, *n* = 46; and Engineering, *n* = 30. Faculty-based comparisons and covariate-adjusted estimates are therefore treated as sensitivity analyses rather than definitive evidence.

### 2.2 Procedure

Data were collected using a web-based questionnaire administered through Google Forms. Data collection commenced immediately after ethical approval had been granted and continued until the end of June 2026. Participation was voluntary, and electronic informed consent was obtained from all participants prior to completing the questionnaire. No financial or other incentives were offered for participation. Ethical approval was obtained from the Social Research Ethics Committee of Izmir Kâtip Çelebi University (Approval No. 2026/07-05, April 1, 2026).

Nine participants did not complete the Future Anxiety Scale. Analyses involving future anxiety therefore used 391 complete cases; descriptive and reliability analyses for the remaining variables used the full sample of 400.

### 2.3 Measures

#### 2.3.1 Mental WellBeing

Mental wellbeing was assessed with the 14-item Warwick–Edinburgh Mental WellBeing Scale (WEMWBS; Tennant et al., 2007), adapted and validated in Turkish by Keldal (2015). Items are rated from 1 (none of the time) to 5 (all of the time), yielding total scores from 14 to 70; higher scores indicate greater mental wellbeing. A representative item is “I've been feeling optimistic about the future.” The scale provides a single total score and does not yield separate Ryff-dimension scores. In the present study, Cronbach's α was 0.92 and McDonald's ω was 0.94.

#### 2.3.2 Future anxiety

Future anxiety was measured with the 5-item Dark Future Scale (DFS; Zaleski et al., 2019), adapted and validated in Turkish by Ergün et al. (2024). Items are rated from 0 (decidedly false) to 6 (decidedly true), yielding total scores from 0 to 30; higher scores indicate greater future anxiety. A representative item is “I am afraid that in the future my life will change for the worse.” In the present study, Cronbach's α was 0.89 and McDonald's ω was 0.93.

#### 2.3.3 Moral orientation

Moral orientation was assessed with the 14-item Moral Orientation Scale (Koçak, 2025). It comprises two separately scored subscales—RMO (8 items) and SMO (6 items)—and no overall moral-orientation total is calculated.

##### 2.3.3.1 Religious moral orientation

The eight-item RMO subscale assesses reliance on religious beliefs and transcendent sources when making moral judgments. Items are rated from 1 (strongly disagree) to 7 (strongly agree), yielding a possible total of 8–56. English translations of representative items are “Being moral requires religion” and “The goodness of a behavior depends on whether it constitutes a religious merit or sin” (Koçak, 2025). Higher scores indicate greater RMO. In the present study, Cronbach's α was 0.93 and McDonald's ω was 0.95.

##### 2.3.3.2 Secular moral orientation

The six-item SMO subscale assesses reliance on reason, human experience, and non-religious ethical principles when making moral judgments. Items use the same 1–7 response format, yielding a possible total of 6–42. English translations of representative items are “Science and reason are sufficient to distinguish right from wrong” and “As long as I live honestly, my religious belief is not very important” (Koçak, 2025). Higher scores indicate greater SMO. In the present study, Cronbach's α was 0.88 and McDonald's ω was 0.92.

### 2.4 Statistical analysis strategy

Primary descriptive, correlation, and moderation analyses were conducted in jamovi (version 2.7.34.0; The jamovi project, Sydney, Australia). Separate moderated regressions were estimated for RMO (Model 1) and SMO (Model 2), with mental wellbeing and each moderator mean-centered before their product term was calculated (Aiken and West, 1991). Unstandardized coefficients and two-sided tests with α = 0.05 are reported. Normal-theory standard errors and *z* tests constituted the primary inferential approach. Interactions were probed at the moderator mean and at ±1 SD. Percentile-bootstrap confidence intervals were based on 10,000 resamples. Sensitivity and verification analyses were independently implemented in Python 3.12.14 using NumPy 2.3.5 and SciPy 1.17.0. Because the RMO interaction was sensitive to estimator choice, interpretation integrates the normal-theory, percentile-bootstrap, and HC3-robust results rather than relying on a single *p* value.

A *post hoc* sensitivity analysis indicated that *n* = 391 provided approximately 80% power (α = 0.05) to detect an interaction of *f* ^2^ = 0.02. This analysis was not an *a priori* sample-size determination and does not exclude smaller true effects; it is reported only to describe the effect-size range the study was positioned to detect (Cohen, 1988).

Observed-variable distributions showed skewness from −0.67 to 0.25 and kurtosis from −0.92 to 1.17. These univariate statistics describe the measured variables but do not by themselves establish normally distributed or homoscedastic regression residuals. In the two primary models, variance inflation factors for the predictor, moderator, and interaction terms ranged from 1.00 to 1.06. Bootstrap intervals and HC3 robust standard errors were therefore used to assess the sensitivity of interaction inferences to distributional and heteroscedasticity concerns.

For the exploratory sociodemographic comparisons reported in Section 3.2, a Bonferroni correction was applied *a priori* to control the familywise Type I error rate across the 36 comparisons conducted (αcorrected = 0.05/36 ≈ 0.0014).

Four sensitivity analyses were undertaken in response to peer review and are reported in Section 3.6: (a) HC3 heteroscedasticity-consistent standard errors; (b) a joint model including both mental wellbeing × RMO and mental wellbeing × SMO interactions; (c) contrasts of the unstandardized and fully standardized joint-model interaction coefficients; and (d) separate moderation models adjusted for gender, age, perceived economic status, and faculty, with both conventional and HC3 inference. Age was mean-centered. Men, participants reporting that their income was more than sufficient, and Theology students served as the reference categories for gender, economic status, and faculty, respectively. Because these analyses were added after the initial submission—and because the Medicine category contained only one participant—their results are treated as supplementary and do not supersede the primary models.

## 3 Results

### 3.1 Preliminary analyses

Table 1 presents descriptive statistics, score reliability, and correlations. Item-level reconstruction confirmed that every stored composite score exactly equaled the corresponding item sum. All four scores showed high internal consistency (α = 0.88–0.93; ω = 0.92–0.95). Mental wellbeing was moderately and inversely correlated with future anxiety (*r* = −0.30, *p* < 0.001). RMO was essentially unrelated to future anxiety (*r* = 0.001, *p* = 0.977), whereas SMO showed a small positive correlation with future anxiety (*r* = 0.16, *p* < 0.01).

**Table 1:** Descriptive statistics, reliability coefficients, and Pearson correlations among the study variables (*N* = 391–400).

Variable	*k*; range	*M*	SD	Cronbach's α	McDonald's ω	1. RMO	2. SMO	3. MWB	4. FA
1. Religious moral orientation (RMO)	8 (8–56)	37.54	12.37	0.93	0.95	–			
2. Secular moral orientation (SMO)	6 (6–42)	21.57	9.64	0.88	0.92	−0.49^**^	–		
3. Mental wellbeing (MWB)	14 (14–70)	51.26	10.28	0.92	0.94	0.16^*^	−0.04	–	
4. Future anxiety (FA)	5 (0–30)	18.68	7.71	0.89	0.93	0.00	0.16^*^	−0.30^**^	–

*k* = number of items. Due to missing data for nine participants on the Future Anxiety scale, descriptive statistics for this scale were computed on *n* = 391; *N* = 400 for the remaining variables. Pearson correlation coefficients are presented below the diagonal. ^*^p < 0.01. ^**^p < 0.001.

### 3.2 Sociodemographic correlates of study variables

Before turning to the primary moderation models, the relationship between the sociodemographic variables measured in the sample and the four scale scores (RMO, SMO, mental wellbeing, future anxiety) was screened in an exploratory fashion (Table 2). As this screening was not based on an *a priori* hypothesis, the findings are treated as descriptive, and a Bonferroni correction (αcorrected = 0.0014) was applied for the 36 comparisons.

**Table 2:** Comparisons between sociodemographic variables and religious moral orientation, secular moral orientation, mental wellbeing, and future anxiety scores (*N* = 391–400).

Sociodemographic Variable	RMO	SMO	MWB	FA
Gender	*t*(223.8) = −1.68, *p* = 0.094	*t*(237.7) = 1.38, *p* = 0.168	*t*(234.1) = 1.86, *p* = 0.064	*t*(233.4) = −5.21, *p* < 0.001^*^
Age (Pearson *r*)	*r* = 0.02, *p* = 0.700	*r* = −0.03, *p* = 0.520	*r* = 0.06, *p* = 0.248	*r* = −0.11, *p* = 0.025
Place where most of life was spent	*F*(3, 396) = 2.68, *p* = 0.046	*F*(3, 396) = 1.38, *p* = 0.249	*F*(3, 396) = 1.99, *p* = 0.116	*F*(3, 387) = 0.06, *p* = 0.979
Current place of residence	*F*(3, 396) = 5.06, *p* = 0.002	*F*(3, 396) = 3.42, *p* = 0.017	*F*(3, 396) = 0.72, *p* = 0.539	*F*(3, 387) = 0.34, *p* = 0.797
Employment status	*F*(2, 397) = 1.51, *p* = 0.222	*F*(2, 397) = 0.06, *p* = 0.939	*F*(2, 397) = 2.79, *p* = 0.063	*F*(2, 388) = 2.83, *p* = 0.060
Perceived economic status	*F*(2, 397) = 1.18, *p* = 0.309	*F*(2, 397) = 2.01, *p* = 0.135	*F*(2, 397) = 10.83, *p* < 0.001^*^	*F*(2, 388) = 7.25, *p* < 0.001^*^
Type of high school	*t*(394.7) = 4.57, *p* < 0.001^*^	*t*(395.8) = −8.05, *p* < 0.001^*^	*t*(364) = −0.20, *p* = 0.846	*t*(374) = 0.68, *p* = 0.498
Faculty	*F*(4, 395) = 20.73, *p* < 0.001^*^	*F*(4, 395) = 43.98, *p* < 0.001^*^	*F*(4, 395) = 0.55, *p* = 0.696	*F*(4, 386) = 3.04, *p* = 0.017
Grade level	*F*(6, 393) = 2.10, *p* = 0.053	*F*(6, 393) = 3.71, *p* = 0.001^*^	*F*(6, 393) = 1.84, *p* = 0.090	*F*(6, 384) = 0.99, *p* = 0.434

Welch-corrected independent-samples *t* tests were used for gender and type of high school; one-way ANOVAs were used for the remaining categorical variables; age was examined using Pearson correlations. These were exploratory screenings. A Bonferroni correction covered 36 tests—(eight categorical variables + age) × four study scores—yielding αcorrected = 0.05/36 ≈ 0.0014. ^*^p < 0.0014. Sparse faculty and grade categories limit the interpretability of those comparisons.

With respect to gender, female participants (*M* = 20.18, SD = 6.97) reported significantly higher future anxiety than male participants (*M* = 15.82, SD = 8.26), *t*(233.4) = −5.21, *p* < 0.001, *d* = 0.58 (a medium effect size); this difference survives the Bonferroni-corrected threshold. No significant gender difference emerged for RMO, SMO, or mental wellbeing (all *p* > 0.06).

Perceived economic status produced a significant differentiation for both mental wellbeing, *F*(2, 397) = 10.83, *p* < 0.001, η^2^ = 0.052, and future anxiety, *F*(2, 388) = 7.25, *p* < 0.001, η^2^ = 0.036 (both differences survive the Bonferroni-corrected threshold). Tukey HSD *post hoc* comparisons showed that participants who perceived their economic status as high (MWB: *M* = 54.89, SD = 7.78; FA: *M* = 16.45, SD = 7.75) reported significantly higher mental wellbeing and lower future anxiety than both the medium (MWB: *M* = 50.52, SD = 10.83, *p* = 0.001; FA: *M* = 19.05, SD = 7.48, *p* = 0.014) and low economic status groups (MWB: *M* = 48.75, SD = 10.65, *p* < 0.001; FA: *M* = 20.37, SD = 7.64, *p* = 0.001); the medium-vs.-low comparison did not reach significance (all *p* > 0.32).

Regarding type of high school, graduates of Imam Hatip (religious vocational) high schools (RMO: *M* = 40.62, SD = 10.82; SMO: *M* = 17.54, SD = 7.95) reported significantly higher RMO, *t*(394.7) = 4.57, *p* < 0.001, *d* = 0.45, and significantly lower SMO than graduates of other high school types (RMO: *M* = 35.18, SD = 12.97; SMO: *M* = 24.65, SD = 9.7), *t*(395.8) = −8.05, *p* < 0.001, *d* = 0.79 (a large effect size). Both differences survived the Bonferroni-corrected threshold, and the observed pattern was consistent with the theoretical expectations underlying the RMO and SMO scales.

Faculty also showed significant differentiation for RMO, *F*(4, 395) = 20.73, *p* < 0.001, η^2^ = 0.174, and SMO, *F*(4, 395) = 43.98, *p* < 0.001, η^2^ = 0.308 (surviving the Bonferroni-corrected threshold). Tukey HSD comparisons showed that Theology (Divinity) students (RMO: *M* = 41.33, SD = 10.52; SMO: *M* = 17.04, SD = 7.28) had numerically higher RMO and lower SMO scores than students in Social and Human Sciences (RMO: *M* = 29.07, SD = 12.94, *p* < 0.001; SMO: *M* = 28.63, SD = 9.12, *p* < 0.001), Health Sciences (RMO: *p* = 0.509; SMO: *p* < 0.001), and Engineering (RMO: *p* = 0.092; SMO: *p* < 0.001). The Medicine category contained only 1 participant, so pairwise comparisons involving this category (all *p* > 0.64) were not interpreted.

Grade level showed a significant differentiation for SMO, *F*(6, 393) = 3.71, *p* = 0.001 (marginally surviving the Bonferroni-corrected threshold). However, because the 0th-, 5th-, and 11th-year categories each contained only 1 participant, this finding should be interpreted with caution, and pairwise comparisons involving these singleton categories were not interpreted. No other sociodemographic variable (place where most of life was spent, current residence, employment status, age) showed a significant difference or association surviving the Bonferroni-corrected threshold (all unadjusted *p* > 0.0014); the uncorrected association between residence and RMO (*p* = 0.002) narrowly missed the corrected threshold and was therefore not interpreted.

### 3.3 Model 1: Religious moral orientation as a moderator

A moderated regression model was estimated with future anxiety as the outcome, mental wellbeing as the predictor, and RMO as the moderator (mean-centered predictors; normal-theory standard errors). As shown in Table 3, mental wellbeing was inversely associated with future anxiety (*b* = −0.250, SE = 0.036, 95% CI [−0.321, −0.178], *z* = −6.85, *p* < 0.001), whereas the RMO main effect was not significant (*b* = 0.040, SE = 0.030, 95% CI [−0.019, 0.100], *z* = 1.33, *p* = 0.183). Under normal-theory inference, the mental wellbeing × RMO interaction was negative (*b* = −0.007, SE = 0.002, 95% CI [−0.012, −0.002], *z* = −2.99, *p* =0.003). The interaction step accounted for an additional 2.0% of outcome variance, Δ*R*^2^ = 0.020, *F*(1, 387) = 8.84, *p* = 0.003, corresponding to *f* ^2^ = 0.023 (Cohen, 1988). Robustness and sensitivity results are reported in Sections 3.5–3.6.

**Table 3:** Moderated regression results for the association between mental wellbeing and future anxiety, with religious moral orientation as moderator (Model 1).

Predictor	*b*	SE	95% CI	*z*	*p*
MWB	−0.250	0.036	[−0.321, −0.178]	−6.85	<0.001
RMO	0.040	0.030	[−0.019, 0.100]	1.33	=0.183
MWB × RMO	−0.007	0.002	[−0.012, −0.002]	−2.99	=0.003

Dependent variable: future anxiety. Estimation method: normal-theory (standard) standard errors. *N* = 391.

Simple slope analyses (Table 4, Figure 1) showed that the inverse association between mental wellbeing and future anxiety was present at all examined levels of RMO and was steeper at high (+1 SD) RMO (*b* = −0.340, *p* < 0.001) than at low (−1 SD) RMO (*b* = −0.160, *p* < 0.001). Because the interaction was estimation-sensitive, these slopes are descriptive probes of the normal-theory model rather than evidence of a robust difference.

**Table 4:** Simple slope estimates for Model 1 by level of religious moral orientation.

Level of RMO	*b*	SE	95% CI	*z*	*p*
Low (−1 SD)	−0.160	0.043	[−0.245, −0.076]	−3.72	<0.001
Average	−0.250	0.036	[−0.321, −0.178]	−6.85	<0.001
High (+1 SD)	−0.340	0.051	[−0.440, −0.240]	−6.65	<0.001

The table shows the simple slopes of mental wellbeing on future anxiety at different levels of religious moral orientation.

**Figure 1:**
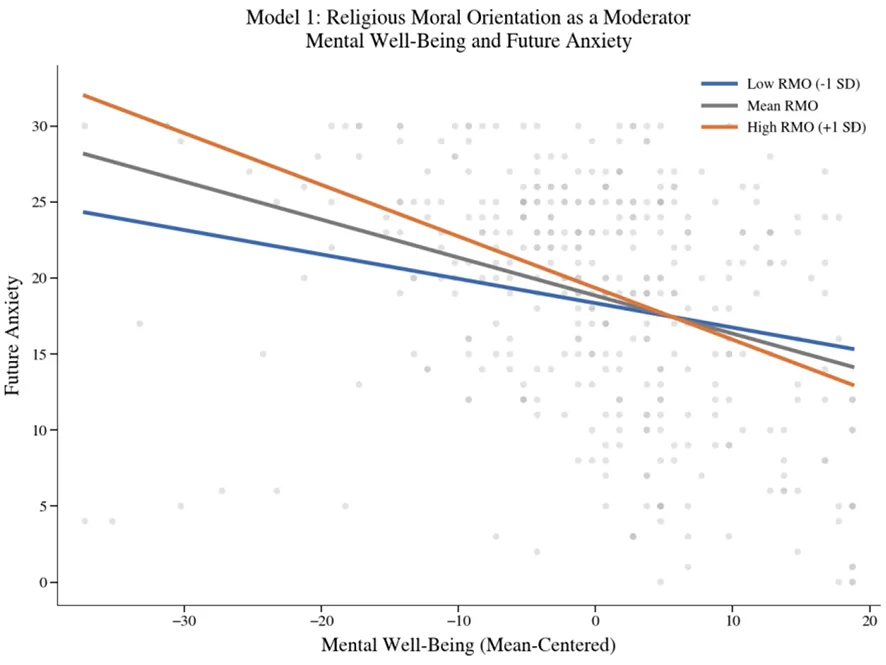
Association between mental wellbeing and future anxiety at low, average, and high levels of religious moral orientation (Model 1).

### 3.4 Model 2: Secular moral orientation as a moderator

A second moderated regression model was estimated with SMO as the moderator. As shown in Table 5, mental wellbeing was inversely associated with future anxiety (*b* = −0.225, SE = 0.035, 95% CI [−0.295, −0.156], *z* = −6.35, *p* < 0.001), and SMO had a positive main effect (*b* = 0.115, SE = 0.038, 95% CI [0.041, 0.189], *z* = 3.03, *p* = 0.002). The mental wellbeing × SMO estimate was negative but did not reach the conventional significance threshold (*b* = −0.006, SE = 0.003, 95% CI [−0.012, 0.001], *z* = −1.76, *p* = 0.078). The interaction step accounted for an additional 0.7% of variance, Δ*R*^2^ = 0.007, *F*(1, 387) = 3.08, *p* = 0.080 (*f* ^2^ = 0.008; Cohen, 1988). It is therefore not interpreted as confirmed moderation.

**Table 5:** Moderated regression results for the association between mental wellbeing and future anxiety, with secular moral orientation as moderator (Model 2).

Predictor	*b*	SE	95% CI	*z*	*p*
MWB	−0.225	0.035	[−0.295, −0.156]	−6.35	<0.001
SMO	0.115	0.038	[0.041, 0.189]	3.03	=0.002
MWB × SMO	−0.006	0.003	[−0.012, 0.001]	−1.76	=0.078

Dependent variable: future anxiety. Estimation method: normal-theory (standard) standard errors. *N* = 391.

For descriptive purposes, simple slopes (Table 6, Figure 2) followed a pattern similar to Model 1—weaker at low SMO (*b* = −0.169, *p* < 0.001) and stronger at high SMO (*b* = −0.282, *p* < 0.001)—but because the interaction term did not reach significance (*p* = 0.078), this pattern should not be interpreted as confirmed moderation.

**Table 6:** Simple slope estimates for Model 2 by level of secular moral orientation.

Level of SMO	*b*	SE	95% CI	*z*	*p*
Low (−1 SD)	−0.169	0.046	[−0.259, −0.079]	−3.68	<0.001
Average	−0.225	0.036	[−0.295, −0.156]	−6.33	<0.001
High (+1 SD)	−0.282	0.050	[−0.380, −0.184]	−5.64	<0.001

The table shows the simple slopes of mental wellbeing on future anxiety at different levels of secular moral orientation.

**Figure 2:**
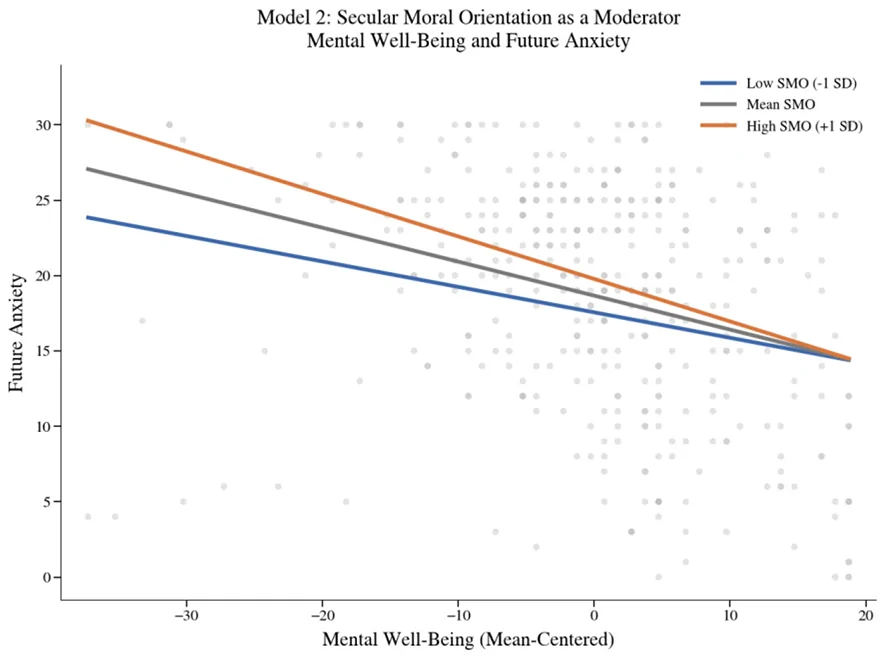
Association between mental wellbeing and future anxiety at low, average, and high levels of secular moral orientation (Model 2).

### 3.5 Robustness check: bootstrap confidence intervals

Because the primary models used normal-theory standard errors, both interaction coefficients were re-estimated with non-parametric percentile bootstrapping (10,000 resamples). For Model 1, the bootstrap 95% CI for the mental wellbeing × RMO interaction was [−0.0136, 0.0002], narrowly including zero. For Model 2, the bootstrap 95% CI for the mental wellbeing × SMO interaction was [−0.0143, 0.0037], consistent with the non-significant normal-theory result.

The SMO interaction was non-significant under both normal-theory and bootstrap inference. For RMO, the coefficient remained negative but the bootstrap interval narrowly included zero, unlike the normal-theory interval. The source of this difference cannot be determined from the interval comparison alone. Accordingly, the evidence for RMO moderation is described as estimation-sensitive and provisional rather than as a robustly confirmed effect.

### 3.6 Sensitivity analyses: robust standard errors, a joint model, and covariate adjustment

HC3 heteroscedasticity-consistent standard errors were computed for both separate moderation models. The mental wellbeing × RMO coefficient remained negative, but its confidence interval narrowly included zero (*b* = −0.0072, robust SE = 0.0037, 95% CI [−0.0145, 0.0000], *t*(387) = −1.96, *p* = 0.0503). The SMO interaction was also non-significant with HC3 inference (*b* = −0.0059, robust SE = 0.0048, 95% CI [−0.0153, 0.0036], *t*(387) = −1.22, *p* = 0.224). Considered with the bootstrap intervals, these results do not provide unambiguous evidence of moderation.

A joint moderated regression included mental wellbeing, both moderator main effects, and both interaction terms. With conventional standard errors, the mental wellbeing × RMO interaction was negative (*b* = −0.0068, SE = 0.0025, 95% CI [−0.0117, −0.0019], *p* = 0.007), whereas the mental wellbeing × SMO interaction did not reach the conventional threshold (*b* = −0.0062, SE = 0.0034, 95% CI [−0.0129, 0.0005], *p* = 0.071). HC3 inference yielded *p* = 0.042 for the RMO interaction and *p* = 0.192 for the SMO interaction. The unstandardized coefficient contrast was non-significant (difference = −0.0006, SE = 0.0038, *t* = −0.16, *p* = 0.877). Because the subscales have different score ranges, a fully standardized joint model was also examined; the interaction coefficients were β = −0.112 for RMO and β = −0.080 for SMO, and their contrast was likewise non-significant (difference = −0.032, SE = 0.054, *t* = −0.59, *p* = 0.553). Thus, significance in one model and non-significance in another does not establish different moderating effects.

Finally, the separate models were re-estimated with gender, age, perceived economic status, and faculty as covariates. With conventional standard errors, the interaction coefficients remained negative (RMO: *b* = −0.0059, SE = 0.0023, 95% CI [−0.0105, −0.0013], *p* = 0.012; SMO: *b* = −0.0065, SE = 0.0032, 95% CI [−0.0129, −0.0002], *p* = 0.045). With HC3 inference, neither adjusted interaction reached significance (RMO: *p* = 0.084; SMO: *p* = 0.155). The SMO interaction therefore crossed the conventional threshold only in one *post hoc* specification. These adjusted results are sensitivity evidence rather than confirmation, and they do not eliminate residual confounding, especially given the singleton Medicine category. Full interaction estimates are summarized in Table 7.

**Table 7:** Sensitivity and verification estimates for the interaction terms.

Analysis	Moderator	Estimator	*b*	SE	95% CI	*p*
Separate	RMO	Normal theory	−0.0072	0.0024	[−0.0120, −0.0025]	0.003
Separate	RMO	Percentile bootstrap	−0.0072	0.0035	[−0.0136, 0.0002]	–
Separate	RMO	HC3	−0.0072	0.0037	[−0.0145, 0.0000]	0.0503
Separate	SMO	Normal theory	−0.0059	0.0033	[−0.0124, 0.0007]	0.078
Separate	SMO	Percentile bootstrap	−0.0059	0.0046	[−0.0143, 0.0037]	–
Separate	SMO	HC3	−0.0059	0.0048	[−0.0153, 0.0036]	0.224
Joint	RMO	Conventional	−0.0068	0.0025	[−0.0117, −0.0019]	0.007
Joint	RMO	HC3	−0.0068	0.0033	[−0.0133, −0.0002]	0.042
Joint	SMO	Conventional	−0.0062	0.0034	[−0.0129, 0.0005]	0.071
Joint	SMO	HC3	−0.0062	0.0047	[−0.0155, 0.0031]	0.192
Adjusted	RMO	Conventional	−0.0059	0.0023	[−0.0105, −0.0013]	0.012
Adjusted	RMO	HC3	−0.0059	0.0034	[−0.0126, 0.0008]	0.084
Adjusted	SMO	Conventional	−0.0065	0.0032	[−0.0129, −0.0002]	0.045
Adjusted	SMO	HC3	−0.0065	0.0046	[−0.0155, 0.0025]	0.155

*b* values are unstandardized. Bootstrap estimates are based on 10,000 resamples; inference is based on the percentile confidence interval, so a separate *p* value is not reported. HC3 confidence intervals and *p* values use t inference. Adjusted models include age, gender, perceived economic status, and faculty. Model *R*^2^ values were 0.114 (separate RMO), 0.118 (separate SMO), 0.150 (joint), 0.217 (adjusted RMO), and 0.220 (adjusted SMO).

## 4 Discussion

The most consistent result was an inverse association between mental wellbeing and future anxiety. Evidence that RMO conditioned this association was less stable: the interaction was negative under normal-theory estimation, but the bootstrap interval included zero and the separate-model HC3 result was *p* = 0.0503. The SMO interaction was non-significant in the primary model. Neither the unstandardized nor the standardized joint-model contrast showed that the RMO and SMO interaction coefficients differed. In the covariate-adjusted models, both interactions crossed the conventional threshold only with conventional—not HC3—standard errors. The data therefore do not support a claim that the two orientations have distinct moderating effects. At the zero-order level, RMO was essentially unrelated to future anxiety, whereas SMO had a small positive association; neither pattern identifies a causal mechanism.

The RMO pattern is compatible with—but does not demonstrate—the possibility that stable religious moral frameworks are associated with how mental wellbeing relates to future-oriented worry. Because mental wellbeing, moral orientation, and future anxiety were measured at the same time, temporal ordering is unknown and reverse or reciprocal associations remain plausible.

One possible interpretation is that RMO provides some individuals with an organized framework of meaning, values, and commitment through which uncertainty is understood (Pargament, 1997; Park, 2010; Weber and Pargament, 2014). This interpretation is consistent with work on religion and uncertainty (Türk et al., 2025) and with research suggesting that commitment to either religious or non-religious worldviews can be associated with adjustment (Galen and Kloet, 2011; Weber et al., 2012). Positive religious attitudes have also been linked to greater happiness among Turkish university students (Francis et al., 2017). It should nevertheless be treated as a hypothesis for longitudinal or experimental research, because meaning, coping, and worldview coherence were not measured as mediators in the present study.

The discussion of virtues by Ayten (2015) potentially supportive resources offers related conceptual background, but the present results do not show that RMO reduces anxiety or increases the effectiveness of mental wellbeing. They show only that the cross-sectional mental wellbeing–future anxiety slope varied across RMO scores under some estimators.

Prior studies have examined religion-related constructs as moderators in occupational stress, chronic illness, and interpersonal adversity (Pirutinsky et al., 2012; Achour et al., 2016; Atlas and Hart, 2023; Hong et al., 2024). The present study extends that question to moral orientation, which concerns normative reference systems rather than religiosity or coping alone (Koçak, 2025). Given the small and estimator-sensitive interaction, however, the present evidence should be viewed as hypothesis-generating rather than as confirmation of a general buffering process.

The findings also require a balanced interpretation of secularity. RMO and SMO are separately scored orientations, not opposite ends of one scale, and respondents may endorse both to varying degrees (Koçak, 2025). Research on non-religious wellbeing emphasizes identity clarity, social support, cultural context, and access to secular sources of meaning (Galen, 2018; Speed and Hwang, 2019; Łowicki and Barker, 2025; Uzarevic and Coleman, 2021). Accordingly, the primary non-significant SMO interaction should not be interpreted as evidence that secular moral frameworks lack psychological relevance or that religious frameworks are generally superior.

### 4.1 Limitations and future directions

Several limitations constrain interpretation. First, the cross-sectional design precludes causal and temporal conclusions. The study was not preregistered, and the power analysis was a *post hoc* sensitivity analysis rather than an *a priori* sample-size calculation. Second, the convenience sample came from one Turkish university and was predominantly female, limiting generalizability. University years are also a period of religious and identity change for many emerging adults (Stoppa and Lefkowitz, 2010); instability in worldview commitment may have affected both RMO and SMO scores. Replication should include non-student and older samples, multiple cultural settings, and direct measures of religious or non-religious identity certainty and change.

Third, all variables were self-reported, creating potential common-method and social-desirability bias. The small RMO interaction (Δ*R*^2^ = 0.020; *f* ^2^ = 0.023) was not supported consistently across estimation methods, the SMO interaction was non-significant in the primary model, and neither the unstandardized nor fully standardized joint-model contrast was significant. Covariate-adjusted models were *post hoc*; their interaction terms were non-significant with HC3 inference, and sparse faculty and grade categories reduce their stability. Future studies should use preregistered covariates, larger and more balanced samples, longitudinal designs, residual diagnostics, and complete coefficient reporting with robust intervals.

Finally, the study did not measure the proposed explanatory processes—such as meaning in life, worldview coherence, identity clarity, coping, or social support. Longitudinal and experimental designs that measure these processes directly are needed before mechanisms or practical effects can be inferred.

### 4.2 Practical implications

The results do not provide a basis for clinical recommendations or for privileging one moral orientation. At most, they suggest that researchers and practitioners may consider clients' self-defined moral or existential frameworks as contextual information when relevant to the client, without assuming that a religious or secular orientation is inherently more adaptive. Any intervention implication requires replication with longitudinal or experimental designs and clinically relevant outcomes.

## 5 Conclusions

Mental wellbeing was inversely associated with future anxiety in this sample. The RMO interaction was negative but sensitive to the inferential method, the SMO interaction was non-significant in the primary model, and neither the unstandardized nor fully standardized joint-model contrast showed that the two interaction coefficients differed. Covariate adjustment did not yield significant interaction terms under HC3 inference. The study therefore identifies a provisional question about moral orientation as a possible boundary condition rather than establishing distinct religious and secular moderation effects.

Replication with preregistered hypotheses, more diverse samples, standardized joint models, and longitudinal data is needed to determine whether the pattern is reliable and to test the roles of worldview coherence, meaning, identity, and social support.

## Data Availability

The datasets presented in this article are not readily available because the datasets contain de-identified participant data collected under ethics approval and informed consent that did not include unrestricted public data sharing. To protect participant confidentiality and privacy, the datasets are not publicly available. They may be made available by the corresponding author upon reasonable request, subject to approval by the relevant ethics committee and applicable institutional regulations. Requests to access the datasets should be directed to Abdurrahman Akbolat Email: abdurrahman.akbolat@ikcu.edu.tr.
